# Role of ion channels in regulating Ca^2+^ homeostasis during the interplay between immune and cancer cells

**DOI:** 10.1038/cddis.2015.23

**Published:** 2015-02-19

**Authors:** T Bose, A Cieślar-Pobuda, E Wiechec

**Affiliations:** 1 Leibniz-Institute of Neurobiology, Brenneckestrasse 6, D-39 Magdeburg, Germany; 2 Department of Clinical and Experimental Medicine, Division of Cell Biology & Integrative Regenerative Medicine Center (IGEN), Linköping University, 581 85 Linköping, Sweden; 3 Biosystems Group, Institute of Automatic Control, Silesian University of Technology, Akademicka 16, 44-100 Gliwice, Poland

## Abstract

Ion channels are abundantly expressed in both excitable and non-excitable cells, thereby regulating the Ca^2+^ influx and downstream signaling pathways of physiological processes. The immune system is specialized in the process of cancer cell recognition and elimination, and is regulated by different ion channels. In comparison with the immune cells, ion channels behave differently in cancer cells by making the tumor cells more hyperpolarized and influence cancer cell proliferation and metastasis. Therefore, ion channels comprise an important therapeutic target in anti-cancer treatment. In this review, we discuss the implication of ion channels in regulation of Ca^2+^ homeostasis during the crosstalk between immune and cancer cell as well as their role in cancer progression.

## Facts

- Ion channels regulate Ca^2+^ influx and downstream signaling pathways in immune and cancer cells.
- Altered regulation of ion channels is implicated in carcinogenesis.
- Cytotoxicity of immune cells against cancer cells depends highly on Ca^2+^ signaling
- Ion channels comprise an attractive tool for targeted therapy for cancer

## Open Questions

- Are blockers of K^+^ and CRAC channels able to inhibit cancer progression?
- What is the role of immune cell-specific ion channels in cancer therapy?
- What cancer-specific ion channels are involved in neoplastic transformation *in vivo*?

Physiological processes depend on the continued flow of ions into and out of cells defeating a barrier impermeable to ions such as plasma membrane, which is built in a form of phospholipid bilayer. Thus, the hydrophobic membrane acts as a serious energy barrier for transporting ions. Ions are charged molecules that have low solubility in the hydrocarbon core of lipid bilayer, thereby having low permeability coefficients across the bilayer. There is a large difference in the electric potential between the two sides of a biological membrane. In order to transfer ions across the membrane and equilibrate both sides of the membrane, eukaryotic cells are equipped in the integrally embedded pore-forming membrane proteins (ion channels) and biological pumps. Such structure allows for the passage of ions through the channel. Opening and closing of the ion channel is usually controlled chemically or mechanically. Depending on the type of ion channel, its conformational change may occur because of changes in the membrane potential (voltage-gated channels), ligand binding (chemical activation) or ligand-driven stretching of the membrane (stretch-activated ion channels). Body response to the external stimuli can be linked to the regulation of ion channel activity. Ion channels play a crucial role in various physiological processes including flow of nerve impulses, muscle contraction, cell division and hormone secretion.^1^ The intracellular concentration of the key signaling ion such as calcium (Ca^2+^) depends on electrical gradients driven in turn by sodium (Na^+^) and potassium (K^+^) channels. The role of ion channels in pathogenesis of various diseases including cancer and its treatment has been extensively studied. The prime function of an immune cell is to remove cancer cells from the body by cytotoxic T lymphocytes (CTL or CD8^+^ cells) and natural killer (NK) cells through polarized discharge of the contents of cytotoxic granules towards the target cells.^2^ The effector function of CTL and NK cells as well as their proliferation and apoptosis of cancer cells largely depend on Ca^2+^ signaling. The role of ion channels in the regulation of intracellular Ca^2+^ concentration is well described in the literature. Alterations in Ca^2+^ homeostasis due to ion channel dysfunction contribute to the common traits of neoplastic transformation, which are known as hallmarks of cancer. These hallmarks include different stages of tumor development like unlimited replication, tissue invasion and metastasis, evasion of apoptosis, sustained angiogenesis, self-sufficiency in growth signals and insensitivity to anti-growth signals.^3, 4^ Additionally, modulation of ion channel-mediated Ca^2+^ concentration in CTLs regulates their antitumor action.^5, 6^

## Regulation of Intracellular Ca^2+^ Concentration

Na^+^ and K^+^ are the most abundant cations in biological systems. Na^+^ ions are mainly present at high concentrations outside the cell, unlike K^+^ ions that are present at high concentrations inside the cell. Gradients for these ions across the cell membrane provide the energy source for action potentials generated by opening of Na^+^ and K^+^ channels^7, 8^ and for transporting solutes and other ions across the cell membrane via coupled transporters. Among several ions, the gradient for Ca^2+^ ions is the largest. The cytosol is surrounded by two big Ca^2+^ stores: the extracellular space, where the Ca^2+^ concentration is ~1.8 mM, and the sarco-endoplasmic reticulum, where the Ca^2+^ concentration varies from 300 *μ*M to 2 mM.^9^ In immune cells, the intracellular Ca^2+^ concentration is ~0.1 *μ*M in the resting state, but it is significantly increased (~10-fold) when the cells are activated.^10^

Plasma membrane Ca^2+^ channels and Ca^2+^ influx are particularly important at different steps of the cell-cycle progression and proliferation of immune cells.^11, 12, 13^ The molecular features of Ca^2+^ channels are well defined, which allows for the distinction of four main types of these channels including voltage-activated, receptor-activated, store-operated and second messenger-operated channels. Receptor-activated, store-operated and second messenger-operated channels are ubiquitous, whereas voltage-activated calcium channels are specific for excitable cells. Voltage-activated calcium channels (e.g., L-, T-, N-, P-, Q-type Ca^2+^ channels) open when the plasma membrane is depolarized. Receptor-activated calcium channels (e.g., P2X purinergic receptors) open when a ligand binds to the channel,^14^ whereas store-operated calcium channels (e.g., transient receptor potential (TRP)) and archetype calcium release-activated channels (CRAC) are activated when the level of Ca^2+^ within the lumen of the ER decreases below a threshold level.^15, 16^ Another type, second messenger-operated channels (e.g., arachidonic acid-regulated Ca^2+^ current) are activated by intracellular second messengers like arachidonic acid.^17^ The role of CRAC, TRPM4 and P2X channels are important in case of immune cells in the continuous effort to keep Ca^2+^ at an optimal level in order to maintain the cellular functions in parallel with ion pumps like Na^+^/K^+^ pumps.^18, 19^ In non-excitable cells including immune cells, the membrane potential plays an important role in setting the electrical driving force for Ca^2+^ entry. In cells where voltage-independent Ca^2+^ channels like TRPM4 and two-pore K^+^ channels (K_2P_) are present, Ca^2+^ influx only depends on the electrochemical gradient over the membrane and intensifies when the membrane potential is more negative (hyperpolarized).^20^

Among different ion channels involved in the regulation of Ca^2+^ homeostasis, CRAC channels are the most important. CRAC channels have been widely characterized^21^ and are known because of their high ion selectivity for Ca^2+^ and low conductance. CRAC channels are activated through the binding of the endoplasmic Ca^2+^ depletion sensor, known as stromal interaction molecule 1 (STIM1) and STIM2 to the CRAC channel units ORAI1-3 (also known as CRACM1-3).^10^ ORAI1 is a widely expressed surface glycoprotein with four predicted transmembrane domains, intracellular amino- and carboxyl-termini and no sequence homology to other ion channels except for its homologues ORAI2 and ORAI3.^22, 23^ The activation of ORAI/CRAC channels involves a complex series of coordinated steps, during which STIM proteins sense the depletion of ER Ca^2+^ stores and pass on this store depletion to the CRAC channels.^24, 25^ In resting cells with filled up Ca^2+^ stores, STIM proteins are diffusely distributed all over the ER membrane. Following the depletion of Ca^2+^ stores, STIM proteins get activated, oligomerize and redistribute into puncta within junctional ER sites, which are in close proximity to the plasma membrane.^26^

## Role of Ion Channels in Maintaining the Normal Membrane Potential

**Figure 1:**
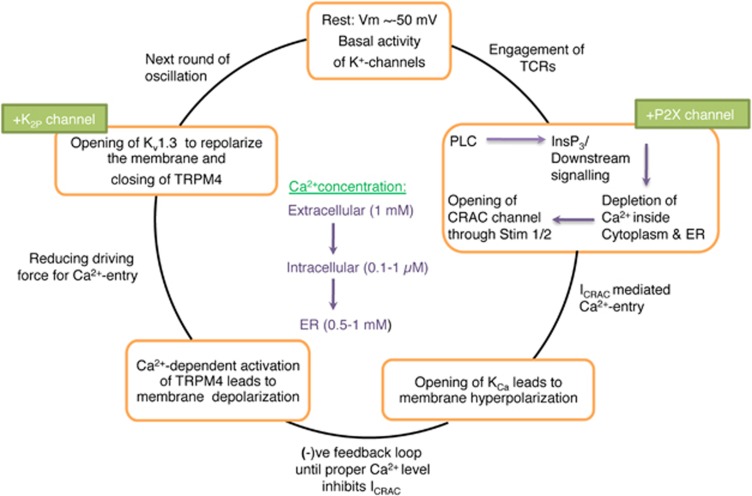
Fluctuations of membrane potential during activation of immune cells. Ca^2+^ influx in lymphocytes depends on the gradient between the extracellular Ca^2+^ concentration (~1 mM) and the intracellular Ca^2+^ concentration (~0.1 *μ*M) as well as the electrochemical gradient established by K^+^ channels (specifically, K_v_1.3, K_ca_3.1 and partially by K_2P_ channels) and the Na^+^-permeable channel TRPM4. CRAC channels are activated upon the engagement of antigen receptors (i.e., TCRs, BCRs). This is mediated through the activation of PLC*γ*, the production of IP_3_ and the release of Ca^2+^ from ER Ca^2+^ stores. The subsequent activation of STIM1 and STIM2 results in the opening of ORAI1 CRAC channels and SOCE. Sustained Ca^2+^ entry through CRAC channels leads to the activation of Ca^2+^-dependent enzymes and transcription factors, including calcineurin and NFAT.^28^ Additionally, P2X receptors (e.g., P2X4 and P2X7) are non-selective Ca^2+^ channels activated by extracellular ATP mediating Ca^2+^ influx in order to augment SOCE-mediated activation of signaling molecules (according to Launay P, 2004; Feske S, 2012). Abbreviations: TCR, T cell receptor; PLC*γ*1, phospholipase C*γ*1; NFAT, nuclear factor of activated T cells; CRAC, calcium release-activated channels; STIM1/2, stromal interaction molecule 1/2; SOCE, store-operated calcium entry; P2X, purinergic receptor 2X

The resting potential of a lymphocyte membrane is ~−50 mV. Membrane potential alterations mainly occur when lymphocytes get activated. TCR engagement activates PLC*γ*1, which catalyzes the hydrolysis of phosphatidylinositol 4,5-bisphosphate (PIP_2_) into inositol trisphosphate (IP_3_) and di-acyl glycerol. IP_3_ stimulates the release of Ca^2+^ from intracellular ER stores, which triggers the opening of plasma membrane CRAC channels. It is the resulting influx of extracellular Ca^2+^ that is responsible for the sustained rise in cytoplasmic Ca^2+^ after TCR stimulation. Ca^2+^ binds to the cytoplasmic Ca^2+^-dependent protein calmodulin, which then activates the phosphatase calcineurin. This phosphatase dephosphorylates and activates the nuclear factor of transcription of activated T cells (NFAT), which enters the nucleus and helps to initiate interleukin-2 (IL-2) gene transcription.^10^ During the activation of immune cells, opening of CRAC channels raises the intracellular Ca^2+^ level. To maintain the balance in membrane conductance, K_Ca_ channels get opened to hyperpolarize the membrane, which results in Ca^2+^ efflux. A negative feedback loop is established when the level of Ca^2+^ inside the cell is high enough to inhibit CRAC channels. Beside the Ca^2+^-dependent activation of TRPM4 channels in T cells, there is also involvement of K_v_1.3 channels in order to repolarize the membrane (Figure 1). Along with these conventional ion channels, the K_2P_ TWIK-related acid-sensitive K^+^ channels 1 and 3 (TASK-1/K_2P_3.1 and TASK-3/K_2P_9.1) are known to regulate immune cell effector functions by hyperpolarizing the membrane.^27^

## Ion Channels in Immune Cells

Activation and the effector role of immune cells is dependent on Ca^2+^ influx, which is regulated by a group of ion channels located in the plasma membrane of the cell. The detailed characteristics of certain ion channels and their implication in the cellular functions became possible with the help of ‘gold standard' patch-clamp technique. The role of individual types of ion channels in the physiology of immune cells is briefly presented.

### K^+^ channels

K^+^ channels comprise the major ion channel family expressed in immune cells that regulate important cellular processes including Ca^2+^-mediated cellular proliferation, migration and finally controlling cell volume.^28^ They regulate membrane potential by driving K^+^ efflux resulting in membrane hyperpolarization. From the superfamily of K^+^ channels, immune cells express voltage-gated (K_v_1.3), calcium-activated (K_Ca_3.1), inwardly rectifying potassium channels (K_ir_) and two-pore gated channels (K_2P_).^29^ In regard to the structural diversity of the channels, there are several types like six transmembrane one pore (K_v_) or transmembrane two pore (K_2P_).^29^ K_v_ channels are further subdivided into three conserved gene families: Kv (shaker-like), Ether-a-go-go (EAG) and KCNQ (K_v_7).^30^ In addition, K_Ca_ channels are grouped into big-conductance calcium-activated channels (BK_Ca_ (K_Ca_1.1)), intermediate-conductance calcium-activated channels (IK_Ca_ (K_Ca_3.1)) and small-conductance calcium-activated channels (SK_Ca_ (K_Ca_2.1, K_Ca_2.2, K_Ca_2.3)).^30^

The role of K_v_1.3 and K_Ca_3.1 in mediating the efflux of K^+^ in order to maintain the hyperpolarization of the cell membrane (Figure 1) is well explained in the literature.^27^ K^+^ channels are differently expressed in various subsets of lymphocytes followed by their activation. For example, naïve and regulatory human T cells mainly express K_v_1.3, whereas the expression of K_Ca_3.1 is upregulated upon activation by cognate antigen.^31, 32, 33^ Interestingly, a recent study has shown that K_v_1.3 channels are indispensable for the differentiation of CD8^+^ T cells into effector cells with cytotoxic ability.^34^ Moreover, K_v_1.3 channels accumulate specifically at the immune synapse (IS) between cytotoxic and target cells in order to modulate the killing process mediated by CTL and NK cells.^35, 36^ In addition, blocking of K_Ca_3.1 in NK cells increases their tumor cell killing ability and comprises an excellent target for cancer immunotherapy.^37^

K_ir_ channels are responsible for stabilization of the resting membrane potential near to the K^+^ equilibrium potential by passing positive charge mostly into the cell (inward direction) rather than in the opposite direction.^38^ This type of channels is present in a significant amount in macrophages, dendritic cells and microglia.^39^ Studies have shown that K_ir_2.0 and K_ir_4.0 family members interact with NIL-16, neuronal variant of interleukin 16 (IL-16).^40^ As the cytokine IL-16 has been characterized mostly in the immune system, the identification of NIL-16 emphasizes the connection of K_ir_ channels with the immune and nervous system. On the basis of the observation that memantine inhibits the amplitude of inwardly rectifying K^+^ current though the K_ir_ channels in macrophages and microglial cells, it is postulated that blocking the K_ir_ channels may influence the functional activity of macrophages.^41^ K_ir_4.1 channel has been lately also found to be a target of the autoantibody response in a subgroup of persons with multiple sclerosis, which suggests that autoreactive T cells are key to the pathogenesis of this disease.^42^

K_2_P (KCNK), better known as 'leak channels' are important for setting the resting membrane potential. Furthermore, their action is mainly voltage-independent and can be regulated via various stimuli including mechanical stimulation, lipids, G_q_ proteins or muscarine.^27, 43^ TASK-1/K_2P_3.1 and TASK-3/K_2P_9.1, the two functional members of the K_2P_ family are expressed in T lymphocytes and contribute to the modulation of T-cell effector function including interferon-*γ* (IFN-γ) and IL-2 secretion as well as T-cell proliferation. Selective blockade of TASK channels present on T lymphocytes leads to improvement of the experimental autoimmune encephalomyelitis course, a model of multiple sclerosis.^27^

### Transient receptor potential (TRP) channel

Among the superfamily of 28 TRP cation channels,^44^ immune cells mainly express TRPMC and TRPM subfamilies like TRPC-1, 3, 5 and TRPM-2, 4, 7.^45^ These channels have biophysical properties to be non-selective and permeable to several cations like Ca^2+^ and Na^+ 45^. Regulation of intracellular Ca^2+^ concentration is indispensable for lymphocyte activation, and TRP channels may both increase Ca^2+^ influx (TRPC3) or decrease Ca^2+^ influx through membrane depolarization (TRPM4). The function of TRPM4 channel is well documented in maintaining the normal membrane potential of an immune cell and controlling the Ca^2+^ flux mechanism.^10^ Interestingly, TRPM4 channel mainly conducts Na^+^ and K^+^ cations.^46^ Activation of TRPM4 channels occurs in response to the increase in intracellular Ca^2+^ concentration resulting in Na^+^ influx, membrane depolarization and a reduction in electrical driving force for Ca^2+^ influx (Figure 1). Therefore, TRPM4 channel acts as a negative feedback mechanism for the regulation of store-operated Ca^2+^ entry by CRAC-ORAI as thereby preventing the cellular Ca^2+^ overload.^47^

### Purinergic receptors

P2X receptors are membrane ion channels with the ability to influx several non-selective cations like Na^+^ and Ca^2+^, and are activated by extracellular adenosine 5'-triphosphate (ATP).^48^ P2X receptors belong to the class of ligand-activated ion channels and there are three P2X receptors expressed in human T cells: P2X-1, 4, 7.^49^ Among these three, principally P2X7 is abundantly expressed in immune cells and regulates Ca^2+^ influx process resulting in the activation of downstream signaling mediators and T-cell proliferation.^50, 51, 52^

### Store-operated calcium channels (SOCs)

CRAC is the major store-operated Ca^2+^ channel of immune cells with the biophysical properties of higher Ca^2+^ dependence and low conductivity in the range of 0.024–0.4 pS.^16^ CRAC channels get opened with the signal of depleting endoplasmic reticulum (ER) Ca^2+^ pool. This signal in ER is mainly mediated by ER Ca^2+^ sensors stromal interaction molecule (STIM) 1 and STIM2 and transferred to the pore-forming subunits of the CRAC channel, mainly ORAI1–3. This results in the activation of the CRAC channel. Lymphocytes express two STIM isoforms, STIM1 and STIM2, which mediate store-operated Ca^2+^ entry in B and T cells.^53, 54^ CD4^+^ and CD8^+^ T cells from ORAI1- and STIM1-deficient patients exhibit defective production of various cytokines, including IL-2, IL-17, IFN-γ and tumor necrosis factor (TNF).^55^ Furthermore, store-operated calcium entry is indispensable for the cytotoxic action of CTLs. STIM1- and STIM2-mediated store-operated calcium entry in CD8^+^ T cells is crucial for anti-tumor immunity.^5^

## Anti-tumor Action of Immune Cells

Human immune system has the great potential to destroy cancer cells either by CTL or NK cells without being toxic to the healthy tissue and organs. These distinct immune cells are able to recognize cancer cell by forming a Ca^2+^-dependent cytotoxic IS with the cancer cell and perform a killing mechanism either through the release of lytic granules and granzymes, or by the activation of Fas-FasLigand receptors (known as death receptors).^2^ Efficient CRAC channels and the resulting increase in the cytosolic Ca^2+^ concentration are necessary for adherence to the target cell as well as its recognition.^56^ The adhesion molecule, particularly lymphocyte function-associated antigen 1 (LFA-1) integrin is essential for this process and interacts with Ca^2+^ in diverse ways.^3^ This includes inside-out (transmission of the regulatory signals originating within the cytoplasm to the external ligand-binding domain of the receptor) signaling-based LFA-1 activation or outside-in (transmission of chemical signals into the cell) signaling via LFA-1.^5^ Interaction between CTL and epithelial tumor cell is integrin-dependent and promotes maturation of the cytotoxic IS and modulates anti-tumor CTL response.^56^ Additionally, LFA-1 activation is implicated in mitochondria positioning at the IS in order to control Ca^2+^-influx through CRAC/ORAI Ca^2+^ channels.^57, 58^ It has recently been shown that store-operated Ca^2+^ release driven by ORAI1 is crucial for lytic granule exocytosis in NK cells and CTLs as well as production of cytokines (TNF-*α* and IFN-*γ*) by NK cells.^59^ Furthermore, delineation of the accurate STIM-ORAI1 ratio could be a feature of the killing efficiency of CTL and NK cells.^3^ Ca^2+^ does not directly play a role in the formation of the IS, but it has enormous effect in controlling the duration and kinetics of the cytotoxic IS between killer immune and cancer cell.^2^ Along with the depolarizing nature of cancer cells, Ca^2+^ concentration can also be a marker of the action of a killer T cell. Small fluctuations from the external Ca^2+^ (~1.2 mM) range of a cancerous tissue can indicate the influence of cancer cell killing by CTL or NK cells.^60, 61^

## Ion Channels in Cancer

Ion channels comprise an important factor influencing the formation and development of tumors. Such malignant transformation leads to enhanced proliferation, abnormal differentiation, impaired apoptosis, and finally uncontrolled migration and invasion (Table 1). This is often associated with altered levels of ion channel expression as well as their activity in the mutated cancer cells.^62^ The role of ion channels in pathogenesis of various diseases including cancer and its treatment has been extensively studied. The major types of ion channels implicated in carcinogenesis are presented below.

### Voltage-gated K^+^ channels

#### Shaker-like

Shaker-type of voltage-gated K^+^ channels regulate cell cycle progression by four mechanisms such as controlling membrane potential oscillations, controlling the cell volume dynamics, controlling calcium signaling and promoting malignant growth through the migratory pathway. Influence of voltage-dependent K^+^ channels in the early stages of cancer development confirms the evidence for the overexpression of these channel proteins in cells exposed to chemical carcinogens.^61^ It has been shown that voltage-gated K^+^ channels affect tumor cell proliferation through the regulation of the membrane potential. As an example, overexpression of K_v_1.1 and K_v_1.3 are found in glioma, lymphoma, breast, lung, pancreas and prostate cancer.^49, 63^ Furthermore, K_v_1.3 channel overexpression is also linked with resistance to apoptosis as shown by the upregulation of K_v_1.3 expression in diffuse large B-cell lymphoma and glioma.^64^

#### EAG channels

The EAG subfamily of voltage-gated K^+^ channels is divided into three distinct groups including EAG (EAG1/ K_v_10.1; EAG2/ K_v_10.2), EAG-like K^+^ (ELK) and EAG-related (HERG/ K_v_11.1). EAG1 overexpression has showed tumorigenic potential and poor overall patient survival in multiple cancer types.^65^ Additionally, EAG1 plays a significant role in cell proliferation and tumor angiogenesis.^66^ Another member of the EAG subfamily of voltage-gated K^+^ channels, particularly EAG2, regulates cell volume dynamics important for cell cycle progression and cell proliferation in medulloblastoma.^67^ Similar to EAG1, HERG overexpression is found in brain, breast, gastrointestinal tract, head and neck, kidney, lung, melanoma, ovary, and thyroid cancers.^63^ Moreover, HERG expression correlates with TNF-mediated tumor cell proliferation.^68^

### K_2P_ channels

K_2P_ channels are typically constitutively open as 'leak channels' in order to stabilize the negative membrane potential. A member of this family, K_2P_5.1 (TASK-2 or KCNK5) plays a major role in the regulation of cell volume, which requires the interplay with Ca^2+^ and Cl^-^ channels. This kind of swelling-activated channel is implicated highly in cancer cell physiology.^69^ Overexpression of K_2P_9.1 (TASK-3 or KCNK9) and K_2P_3.1 (TASK-1 or KCNK3) is found in breast, gastrointestinal tract, lung, adrenal cancers and melanoma.^70^ Additionally, overexpression of K_2P_9.1 in breast cancer cell lines promotes tumorigenesis and confers resistance to hypoxia and serum withdrawal.^71^ In general, rapidly proliferating cancer cells are more depolarized in nature with a membrane potential varying from −20 to 40 mV.^72^ Therefore, membrane depolarization plays a functional role in tumor progression inducing DNA synthesis and promoting mitotic activities, which in turn leads to tumor invasion.^73^ As potassium conductance is the major regulatory factor in maintaining relatively depolarized state of the cell, the roles of potassium channel inhibitors in controlling polarization phenomenon of tumor cells remains to be revealed.

### Ca^2+^-activated K^+^ channels

Ca^2+^-activated K^+^ channels are regulated by Ca^2+^ concentration inside the cells. This kind of channels has a major role in cancer metastasis process, which cause >90% of cancer deaths.^74^ Tumor metastasis is a dynamic process involving mobilization of primary tumor cells by migration into other non-tumoral regions. Thus, ion channels are involved in migration, which plays a major role in the initiation of metastasis process.^75^ As an example, BK_Ca_ and SK_Ca_ channels are implicated in metastasis as they have been shown to promote breast cancer cell migration.^76^ Furthermore, SK_Ca_ channels form a complex with the ORAI1 channel for localized calcium entry within lipid rafts in order to enhance cancer cell migration and metastasis.^77^ In general, overexpression of K_ca_1.1 and K_ca_3.1 has been shown in bone, brain, breast, ovary, pancreas cancers and brain, gastrointestinal tract, melanoma and prostate cancers. Interestingly, application of K_ca_1.1 and K_ca_3.1 channel inhibitors decreases the migration of human glioma and experimental transformed renal epithelial cells respectively.^78, 79^

#### K_ir_ channels

As mentioned above, K_ir_ channels allow for easy movement of K^+^ into the cell. They are activated by PIP_2_, but they can also be modulated by other regulatory factors such as ATP (ATP-sensitive K^+^ channels) and G-proteins (G protein-gated K_ir_ channels) or by some non-specific regulators including polyamines, kinases, pH and Na^+^ ions.^80^

The mRNA upregulation of the G-protein regulated inward-rectifier K^+^ (GIRK) channel called K_ir_3.1 (GIRK1) has been shown in invasive breast cancer and non-small-cell lung cancer. Additionally, overexpression of GIRK1 in both types of tumors was correlated with poor prognosis for the patients.^81, 82^

### TRP channels

TRP cation channels have been implicated in various pathological states including cancer due to their role as intracellular Ca^2+^ release channels. Recent studies have shown the association of TRP channels with various cancer types such as melanoma^83^ (TRPM1), prostate cancer^84, 85, 86^ (TRPV2, TRPV6, TRPM8), hepatoblastoma^87^ (TRPV1) and glioblastoma^88, 89^ (TRPC6). Besides the roles of volume control and motility, TRPM8 channel serves as a potential marker for metastatic prostate cancer.^84^ Another TRP channel that has been implicated in enhanced motility and metastasis of cancer cells is TRPM7 channel.^90, 91^ Furthermore, TRP channels are also involved in angiogenesis,^92, 93, 94^ thus their inhibitors might be considered a good pharmaceutical target for cancer therapy. TRPV6, TRPM7 and TRPM8 are also associated with proliferation of breast and prostate cancer cells.^95, 96, 97^ Interestingly, sustained Ca^2+^ flux through TRP channels can itself be a diagnostic marker for a cancer cell and can be inhibited with a TRP channel inhibitor.^98, 99^

## Purinergic Receptors

The ATP-dependent activity of P2X7 channel is associated with various physiological functions including cell proliferation, cell death and cytokine secretion. Recent studies have implicated the role of P2X and P2Y receptors in B cell leukemia,^100^ melanoma and colorectal cancer.^101, 102, 103^ Targeting the P2X7 receptor by selective P2X7 agonists as well as P2X7 antagonists in cancer has shown anti-tumor effect.^101, 104^ Furthermore, the effect of ATP infusion in patients with advanced lung cancer has proven the potential of ATP, which might become an anti-cancer agent in the future.^105, 106, 107, 108^ However, larger studies are required in order to verify these findings.

### Store-operated calcium channels (SOCs)

SOC-mediated sustained increase in the cytosolic Ca^2+^ has shown to trigger apoptosis in tumor cells.^109^ STIM1-ORAI1 driven store-operated calcium entry seems to be indispensable for migration and metastasis of breast cancer, cervical cancer and hepatocarcinoma, which was potently blocked by the store-operated calcium entry inhibitor.^110, 111, 112, 113^ Moreover, CRAC channels are implicated in VEGF-activated Ca^2+^ influx promoting angiogenesis, which might be crucial for cancer progression.^111^

## Ion Channel Modulators

Ion channels are often overexpressed in numerous types of tumors and their altered activity plays a significant role in apoptosis resistance, proliferation and metastasis of cancer cells. Thus, blocking the activity of ion channels seems to be an obvious strategy to impair cancer growth. However, such treatment is not as straightforward as it may look. When targeting ion channels, we aim at efficient killing of cancer cells without causing toxic effects in other tissues expressing the same or related channels. A vast amount of known ion channels blockers are used to treat cardiac arrhythmias or epilepsy (anticonvulsants);^114^ thus, incorporating them into oncology is accompanied by the risk of heart or nervous system disorders.

Unspecificity of ion channel blockers is still a big challenge that needs to be overwhelmed to avoid serious side effects during oncological treatment. Specific inhibition can be obtained by developing monoclonal blocking antibodies, antisense oligonucleotides, small interfering RNAs, peptide toxins and novel small organic compounds.^115^ As discussed by Arcangeli and Becchetti, to improve the efficiency of ion channels targeting cancer, one should also focus on finding inhibitors recognizing conformational changes in ion channels (e.g., open channel *versus* close channel). So far, such an approach was found to be possible in a case of lamotrigine and lidocaine that preferentially target open and inactivated voltage-gated Na^+^ channels, without distinguishing other conformational states.^116^ Similar property exhibits in R-roscovitine recognizing open HERG channel.^117^

Interesting alternative for conventional ways of targeting ion channels in cancer treatment are some dietary compounds.^118^ Curcumin, resveratrol (grape polyphenol), docosahexaenoic acid (omega-3) and epigallocatechin gallate (catechin from green tea) extract were shown to modulate ion channels activity and suppress migration and growth of breast and ovarian cancer cells.^119, 120, 121, 122^ Other examples of targeting ion channels in cancer and immune cells are presented in Table 2.

## Conclusions and Future Perspectives

**Figure 2:**
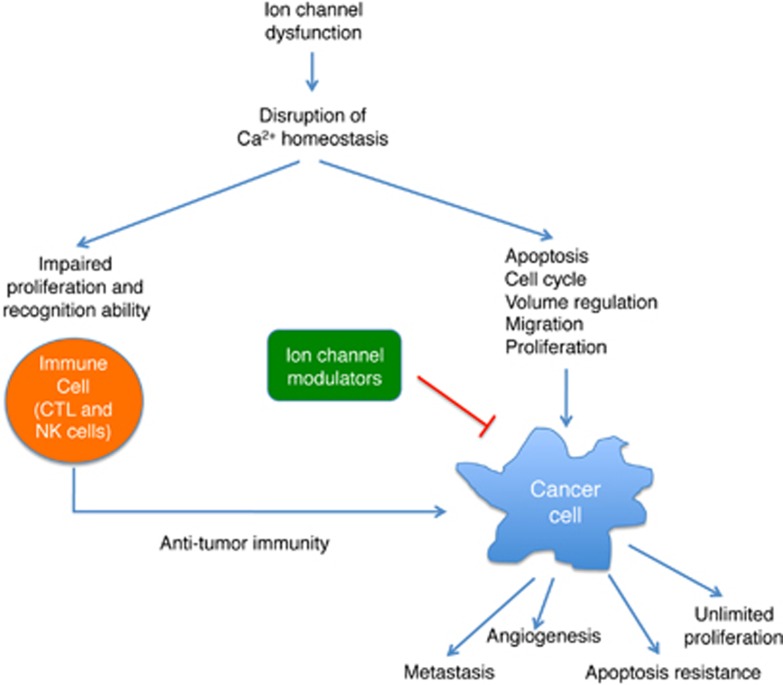
The influence of ion channels on the interaction between the immune system and cancer as well as their role in neoplastic transformation

The main task of the immune system is to defend against attacks by foreign invaders including bacteria, viruses, fungi, parasites and other microorganisms. It has been shown by the researchers from both immunology and oncology fields that cancer cells are also recognized by the immune system, and their proliferation can be controlled immunologically. Alterations in ion channel-based Ca^2+^ signaling are linked to the behavior of cancer cells. Recent studies indicate the significance of ion channels and Ca^2+^ signaling in activation of cancer killing immune cells as well as cancer progression. Generation of an appropriate Ca^2+^ response, which is induced by recognition of a tumor antigen is driven by above-described ion channels (Figure 2). Regulation of certain features of cancer cells by decreasing the activity of ion channel proteins is still under investigation. The market success of Ambien (GABA_A_ receptor inhibitor for the treatment of insomnia) and Norvasc (Ca^2+^ channel blocker used to lower blood pressure and to treat angina pectoris) have energized the drug market to explore more the ion channel field searching for new therapeutics including cancer therapy. Nevertheless, the ion channel-based treatment comprises still far unused anti-cancer strategy. Thus, future research will focus on ion channels as therapeutic target in order to inhibit proliferation of cancer cells and promote their apoptosis together with modulation of cancer-specific cytotoxicity of immune cells. Furthermore, studies involving mutating ion channels in cancer using animal models should uncover novel insights into the ion channel function in tumorigenesis.

## Figures and Tables

**Table 1:** The role of distinct ion channels in cancer development and progression

**Ion channels**	**Expression profile**	**Cancer type**	**References**
*Proliferation of cancer cells*			
Shaker-like K^+^ channels (K_v_1.1, K_v_1.3, K_v_1.5)	Gene and protein upregulation	Glioma, breast cancer, lung cancer, pancreas cancer, prostate cancer, lymphoma	^64, 123^
EAG K^+^ channels (EAG1, EAG2)	Gene and protein upregulation	Medulloblastoma, breast cancer, head and neck cancer, melanoma, gastrointestinal tract cancer	^65, 66, 67^
EAG-related K^+^ channels (HERG/K_v_11.1)	Gene and protein upregulation	Melanoma, neuroblastoma, breast cancer	^68^
Ca^2+^-activated K^+^ channels (K_Ca_3.1)	Gene and protein upregulation	Glioma, breast cancer, ovarian cancer, prostate cancer, melanoma	^124, 125, 126, 127^
TRP (TRPC6, TRPV6, TRPM7, TRPM8)	Gene and protein upregulation	Breast cancer, prostate cancer, head and neck cancer, human glioblastoma cell line	^89, 95, 96, 97, 128, 129^
P2Y (P2Y2), P2X (P2X7), P2U	Gene and protein upregulation	Melanoma, colorectal cancer cells, lung cancer cells	^101, 130, 131^
SOCs (ORAI1/STIM1)	Gene and protein downregulation	Lung cancer cells, cervical cancer	^113, 132^
SOCs (ORAI1/STIM1)	Gene and protein upregulation	Cervical cancer, glioblastoma cells	^113, 133^
			
*Cell migration and metastasis*			
EAG K^+^ channels (EAG1/ K_v_10.1)	Gene and protein upregulation	Migration of breast cancer cells	^134^
Ca^2+^-activated K^+^ channels (KCNMA1, SK3/ORAI1, K_Ca_1.1, K_Ca_3.1)	Gene and protein upregulation	Breast cancer→metastasis to brain Breast cancer→bone metastasis Migration of glioma cells, transformed renal epithelial cells and breast cancer cells	^75, 76, 77, 78, 135^
K_ir_ channels (K_ir_3.1/GIRK1)	Gene and protein upregulation	Primary breast cancer→axillary lymph node metastasis	^81^
TRP (TRPM7, TRPM8, TRPV1, TRPV6)	Gene and protein upregulation	Lung cancer cells, primary breast cancer, prostate cancer cells, squamos carcinoma, hepatoblastoma	^90, 91, 97, 136, 137, 138^
P2X (P2X7)	Gene and protein upregulation	Breast cancer cell line	^139^
SOCs (ORAI1/STIM1)	Gene and protein upregulation	Breast cancer, cervical cancer, hepatocarcinoma, glioblastoma	^111, 112, 113, 140^
			
*Tumor angiogenesis*			
EAG K^+^ channels (EAG1)	Gene and protein upregulation	Breast cancer and other solid tumors	^65, 66^
TRP (TRPC6 )	Gene and protein upregulation	Human glioblastoma cell line	^88, 94, 141^
SOCs (ORAI1/STIM1)	siRNA- or dominant-negative mutant-mediated knockdown	VEGF-induced angiogenesis observed in tumors	^141, 142^
			
*Apoptosis resistance*			
Shaker-like K^+^ channels (K_v_1.3)	Gene and protein upregulation	Large B-cell lymphoma, glioma	^64^
TRP (TRPA1)	Gene and protein upregulation	Lung cancer cell line	^143^
P2X (P2X7)	Gene and protein downregulation	Breast cancer, melanoma	^104^
SOCs (ORAI1)	siRNA-mediated knockdown	Prostate cancer cell line	^109, 144^

**Table 2:** Ion channel blockers in immune and cancer cells

**Ion channel blocker**	**Ion channel**	**Cell type**	**Comments**	**References**
Margatoxin (MgTX) Charybdotoxin (CTX)	Kv1.3	T lymphoctyes, Jurkat cells	Antiproliferative effect in T-lymphoytes, regulation of immunoresponsiveness	^145, 146^
TRAM-34, NS6180, ShK-186	Kv1.3, KCa3.1	NK cells, leukemia cells	Inhibition of KCa3.1 increased the degranulation of adherent NK cells and their ability to kill K562 leukemia cells	^147^
R-roscovitine	Kv1.3, Kv2.1, Kv4.2, HERG (Kv11.1)	Leukemia	Roscovitine is well known cyclin-dependent kinase inhibitor	^148, 149^
mAb56	EAG1 (Kv10.1)	Pancreas carcinoma, breast cancer	Inhibition of tumor cell growth both *in vitro* and *in vivo*.	^150^
Way 123,398	HERG (Kv11.1)	Colorectal cancer	Reduced cell migration of H630, HCT and HCT8 cells; unaffected growth of HEK 293 cells	^151^
Way 123,398; CsCl; E4031	HERG (Kv11.1)	Acute myeloid leukemia	Impaired cell proliferation.	^152, 153^
Cisapride	HERG (Kv11.1)	Gastric cancer	Inhibition of cells entering S phase from G1 phase of the cell cycle.	^154^
Verapamil	ERG (Kv11.1)	Lung cancer, melanoma, colon cancer	Increased survival rate for patients treated with verapamil+chemotherapy	^155, 156^
UNBS0 (Cardenolide)	Na^+^/K^+^ ATPase	Glioblastoma	Decrease in intracellular ATP concentration leads to autophagy in glioma cells UNBS0 shows anti-proliferative activity *in vitro* in 58 human cancer cell lines	^18, 157^
Tetrodotoxin (TTX)	Nav1.5, Nav1.6 Voltage-gated Na^+^ channels	Human melanoma, macrophages, breast cancer	TTX and shRNA knockdown of Nav1.6 has inhibitory effects on both cellular invasion of macrophages and melanoma cells	^158, 159^
Charybdotoxin (CTX)	K_ir_ (IK1)	Human melanoma	Reduced migration of melanoma cells treated with CTX	^160^
Zinc, methanandamide	K_2P_9.1 (TASK-3)	Ovarian cancer	Reduction in cell proliferation and increase in apoptosis	^161^

## Abbreviations

ATP: adenosine 5'-triphosphate
BK_Ca_: big-conductance calcium-activated potassium channel
CRAC: calcium release-activated channels
CTL: cytotoxic T-lymphocyte
IFN-*γ*: interferon gamma
IK_Ca_: intermediate-conductance calcium-activated potassium channel
IL-2: interleukin-2
IL-16: interleukin-16
IS: immune synapse
IP_3_: inositol trisphosphate
K_Ca_: calcium-activated potassium channel
K_ir_: inwardly rectifying potassium channels
K_v_: voltage-gated potassium channel
LFA-1: lymphocyte function-associated antigen 1
NFAT: nuclear factor of activated T cells
NK: natural killer cell
PIP_2_: phosphatidylinositol 4,5-bisphosphate
PLC*γ*1: phospholipase C*γ*1
P2X: purinergic receptor 2X
SK_Ca_: small-conductance calcium-activated potassium channel
SOCE: store-operated calcium entry
STIM1/2: stromal interaction molecule 1/2
TASK1/3: TWIK-related acid-sensitive K^+^ channels 1/3
TCR: T-cell receptor
TNF-*α*: tumor necrosis factor alpha
TRP: transient receptor potential
